# Multi-dimensional evaluation of pain response in low day-age calves to two types of dehorning

**DOI:** 10.3389/fvets.2024.1406576

**Published:** 2024-05-22

**Authors:** Weiguo Cui, Mengyu Liu, Tianyu Gu, Shuai Zhao, Guoan Yin

**Affiliations:** ^1^College of Animal Science and Veterinary Medicine, Heilongjiang Bayi Agricultural University, Daqing, China; ^2^Key Laboratory of Exploration and Innovative Utilization of White Goose Germplasm Resources in the Cold Region of Heilongjiang Province, Daqing, China

**Keywords:** dehorning hot-iron, dehorning cream, acute pain, autonomic response, animal welfare

## Abstract

**Introduction:**

Dehorning calves is necessary to minimize injury because intensive raising circumstances make horned cows more aggressive. However, acute pain is commonly perceived by farm animals when undergoing painful practices such as dehorning, affecting their health status and quality of life. By quantifying the magnitude of pain and discomfort associated with dehorning, we aim to contribute to a more humane and sustainable cattle farming industry.

**Methods:**

The objective of this study was to evaluate the behavioral, physiological, and emotional effects of acute dehorning pain in calves using two methods: dehorning cream and dehorning hot-iron.30 Holstein calves aged 4 days were selected for the study. These calves were randomly assigned to two experimental groups based on the method of disbudding: dehorning cream (*n* = 15) and hot-iron dehorning (*n* = 15). Before and after dehorning, we evaluated their physiological indicators of infrared eye temperature, concentrations of substance P, IL-6, cortisol, haptoglobin, as well as emotional state, and pain-related behavioral reactions.

**Results:**

Post-dehorning, the duration of lying down decreased significantly in both groups (DI and DC: 0–4 h) after dehorning (*p* < 0.05). Both groups exhibited increased frequencies of pain-related behaviors such as head shaking (DI: 1–7 h, DC: 1–6 h), ear flicking (DI: 2–7 h, DC: 2–7 h), head scratching (DI: 2–3 h, DC: 1–7 h), and top scuffing (DI: 2 h, DC: 2–7 h) compared to pre-dehorning (*p* < 0.05). The DC group demonstrated a higher frequency of head-shaking, ear-flicking, head-scratching, and top-rubbing behaviors, along with a longer duration of lying down (0–4 h), compared to the DI group (*p* < 0.05). Post-dehorning, play behavior reduced significantly in both groups (6–8 h) (*p* < 0.05), whereas judgment bias and fear levels showed no significant change (*p* > 0.05). Physiological measures including eye temperature, and blood levels of substance P and IL-6, did not differ significantly between the groups before and after dehorning (*p* > 0.05). However, 48 h after dehorning, calves in the DC group had significantly higher haptoglobin levels compared to the DI group (*p* = 0.015). Additionally, salivary cortisol levels in the DC group increased significantly at 3.5 h and 7 h post-dehorning (*p* = 0.018, *p* = 0.043).

**Discussion:**

Both hot-iron and cream dehorning induced pain in calves, as evidenced by increased pain-related behaviors, elevated salivary cortisol, and higher haptoglobin levels, alongside reduced positive behaviors. Notably, these effects were more pronounced in the DC group than in the DI group, suggesting that dehorning hot-iron may be a comparatively less stressful dehorning method for young calves. Moreover, the brief duration of pain response and weaker response to dehorning observed in 13-day-age calves in this study suggests that dehorning at younger ages may be more advisable and warrants further research.

## 1 Introduction

While dehorning makes horned cows submissive and easier to handle, the intensive feeding approach puts horned cows at risk of attacking humans and other cows (1). It’s important to know when to dehorn calves. Dehorning triggers nerve pain within the calf horn due to the presence of the R. zygomaticotemporal muscle (2). Dehorning performed within 30 days of birth is said to reduce pain because the horn has not fully grown and because of the calf’s immature development (3). However, this does not avoid the perception of acute pain and the behavioral, physiological, and emotional consequences that it can cause. Moreover, productive performance can also be compromised due to the activation of the hypothalamic–pituitary–adrenal axis (4, 5). Consequently, the developmental stage of the calf must be carefully taken into account while determining the best time to dehorn it.

Dehorning cream and dehorning hot-iron are two commonly used techniques. On American farms, the usage of dehorning cream has grown dramatically in recent years (6). Both approaches come with tissue damage and suffering. Dehorning hot-iron produces a strong pain response and may result in brain necrosis and inflammation, endangering the welfare of the calf (7–9). Application of dehorning cream has been associated with severe burns on the heads of calves, leading to increased stress sensitivity (10, 11). Although farms frequently employ non-steroidal anti-inflammatory medications and local anesthetics to reduce post-dehorning pain, the calves nevertheless feel discomforts (12–14).

Determining the effects of various dehorning techniques on calves requires accurate and efficient pain measurement during the dehorning process. It can be challenging to instinctively determine pain in animals, particularly if they have a high pain threshold or do not exhibit clear outward symptoms (15). Accurate pain evaluation requires objective signs, such as physiological markers and behavioral changes. The current International Association for the Study of Pain (IASP) definition of pain as “An unpleasant sensory and emotional experience associated with actual or potential tissue damage, or described in terms of such damage” that the inability to communicate does not negate the possibility that a non-human animal experiences pain, which may favor the exhibition of modifications physiological, emotional and behavioral in the species under study (16, 17). One non-invasive way to evaluate animal welfare and determine the intensity of discomfort is through behavior observation (18). Pain can affect the frequency, intensity, and duration of pain-related behaviors such as lying down, ear-flicking, and head rubbing (11, 19, 20). Studies on pigs have shown a correlation between the frequency of pain-related behaviors and the level of pain (*p* < 0.001) (21). Furthermore, physiological indicators of pain in calves include blood substance P levels, salivary cortisol levels, haptoglobin levels and ocular temperature (22, 23). Animals in pain have been shown to exhibit alterations in cortisol concentrations and a stress-induced glucocorticoid response (24). Measuring substance P and cortisol levels simultaneously can yield a more thorough evaluation of pain intensity. Cattle’s eye temperature can reflect temperature changes brought on by stress that due to infrared thermography is a non-invasive tool that allows evaluating temperature changes in the lacrimal caruncle of the tear caruncle due to stress, pain or illness (25–28).

The question of whether animals can feel emotions has attracted a lot of research, and it is generally accepted that they can feel emotions including fear and pain (29). In animals, the concept of “affective state” or “emotion” refers to their own experiences, emotions, or moods. It is believed to be an external representation of their behavior, physiology, mental processes, and subjective consciousness (30). Notably, animals’ emotional cognition can be affected by pain (15). For example, calf discomfort results in altered attention and biased judgment in addition to decreased play behavior (31, 32). The self-rewarding and endorphin-releasing nature of play behavior makes it a popular test subject for evaluating an animal’s emotional condition. Animal play behavior decreases with a lower happy emotional state (33).

Furthermore, animals’ emotional states are reflected in their reactions to both positive and negative environmental stimuli (34). Their motivation and preferences may be impacted by abnormal affective states, which can also cause aberrations in their subjective consciousness and disturb normal physiological processes (35). Since animals are unable to express their subjective experiences orally, researchers use the cognitive aspects of emotions to measure affective states. For instance, judgment bias tests are accepted methods for assessing animals’ long-term emotional states and how they relate to animal welfare (36). The purpose of this study was to compared the efficacy of various dehorning techniques on calves to identify the method that minimizes pain. This study assumes that the two dehorning methods will have significantly different effects on the behavior, physiology, and emotions of low day-age calves. The findings serve as a foundational framework for refining calf dehorning practices, contributing significantly to the advancement of research on the mitigation of dehorning-related pain and to the enhancement of theoretical constructs underpinning animal welfare.

## 2 Materials and methods

Thirty-five 4-day-age Holstein calves born on the same day from Harbin Wandashan Dairy Cattle Breeding Co. were used in this investigation. The experiment adhered to animal protection guidelines and was approved by the Science and Technology Ethics Committee of Heilongjiang Bayi Agricultural and Reclamation University (approval number: DWKJXY20). The calves were taken away from their mothers as soon as they were born, weighed, tagged, and documented. After that, they were moved to nursery pens. Five calves were eliminated during the training period because they had severe diarrhea judged by professional veterinarian. The remaining 30 calves were randomized into two groups, dehorning hot-iron group (*n* = 15) and dehorning cream group (*n* = 15). The calves were assigned numerical tags, after which the first fifteen unique numbers derived from a random number generator (utilizing the website www.calculator.net) were allocated to the dehorning hot-iron (DI) group. The remaining calves were subsequently assigned to the dehorning cream (DC) group. This method ensured a random distribution of calves across the two dehorning treatment groups.

### 2.1 Feeding management

The calves had three daily feedings of milk at 05:30, 12:30, and 17:30 while living in groups. On the third day after birth, water was given, and it was thereafter freely accessible for 30 min following the last milk feeding. The calves were given libitum concentrate supplement on day seven, which contained the following ingredients: 14% moisture, 22% crude protein, 12% crude fiber, 12% crude ash, 0.4% total phosphorus, 0.5–2.5% calcium, and 0.2–1.5% sodium chloride. Large-scale cattle farm standards were followed in the daily cleaning of bedding polluted with excrement, health checks, temperature records, uniform feeding, immunization, and disease detection processes.

### 2.2 Study training

**Figure 1:**
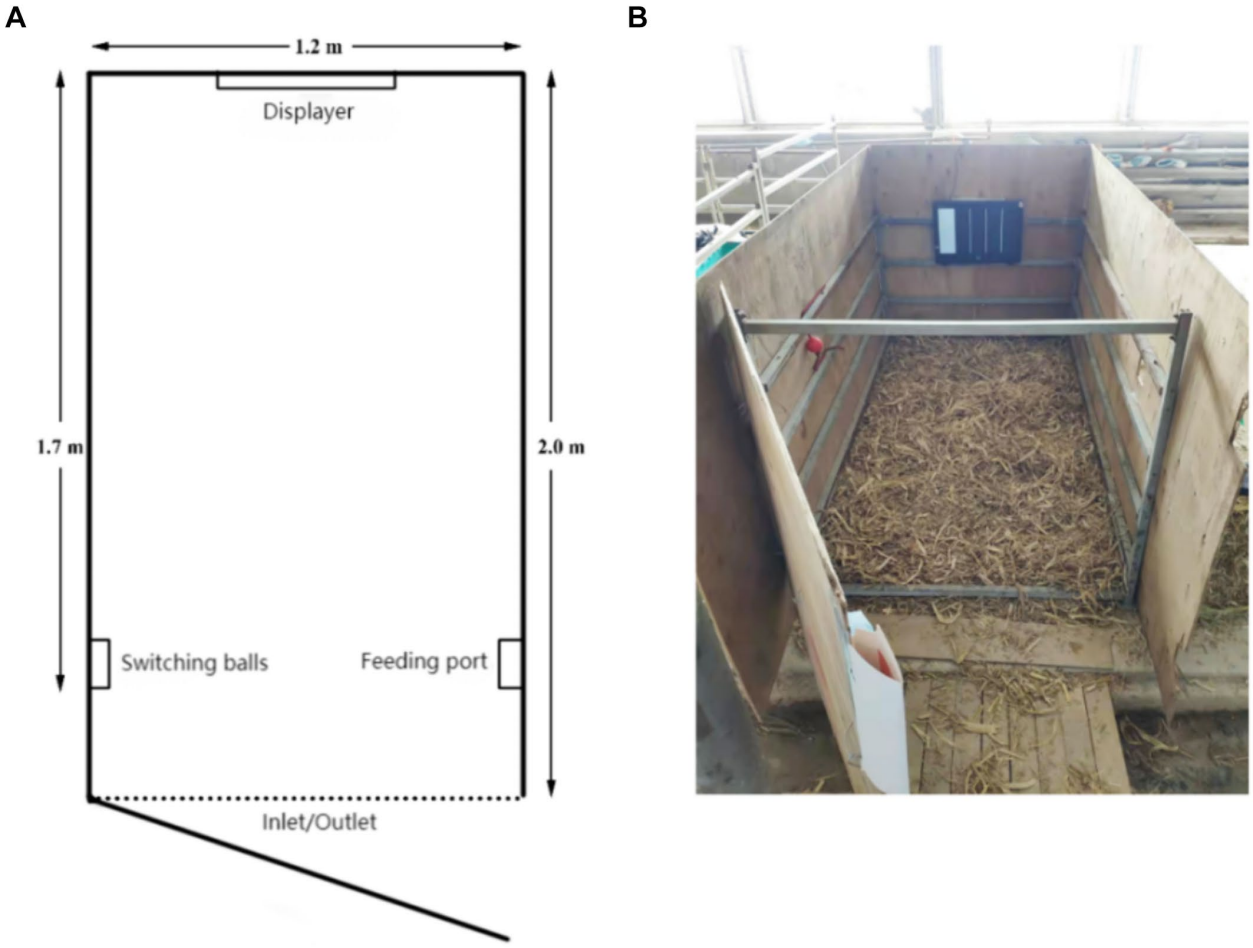
**(A)** Floor plan of the judgment bias training pen. **(B)** Actual view of the judgment bias training pen.

This study’s judgment bias test cattle pen was created using Neave et al.’s design as a model (32). The 200 cm × 140 cm × 150 cm training cage had a steel frame and plywood construction (Figure 1). A PPT flip pen was used to operate a monitor that was positioned across from the enclosure entrance at a height of 50 cm above the floor. On both sides of the fence, switching balls and milk access ports were placed 30 cm from the door and 50 cm from the ground. A monitoring device (JDVISION, AD-132GE, China) was installed in the enclosure to continuously record the activity of the calf during the testing process, guaranteeing that every region was observed. Training and testing bottles (2 L) were sourced from Purina, while training rattles were dog training rattles (Leerburg, United States).

**Figure 2:**
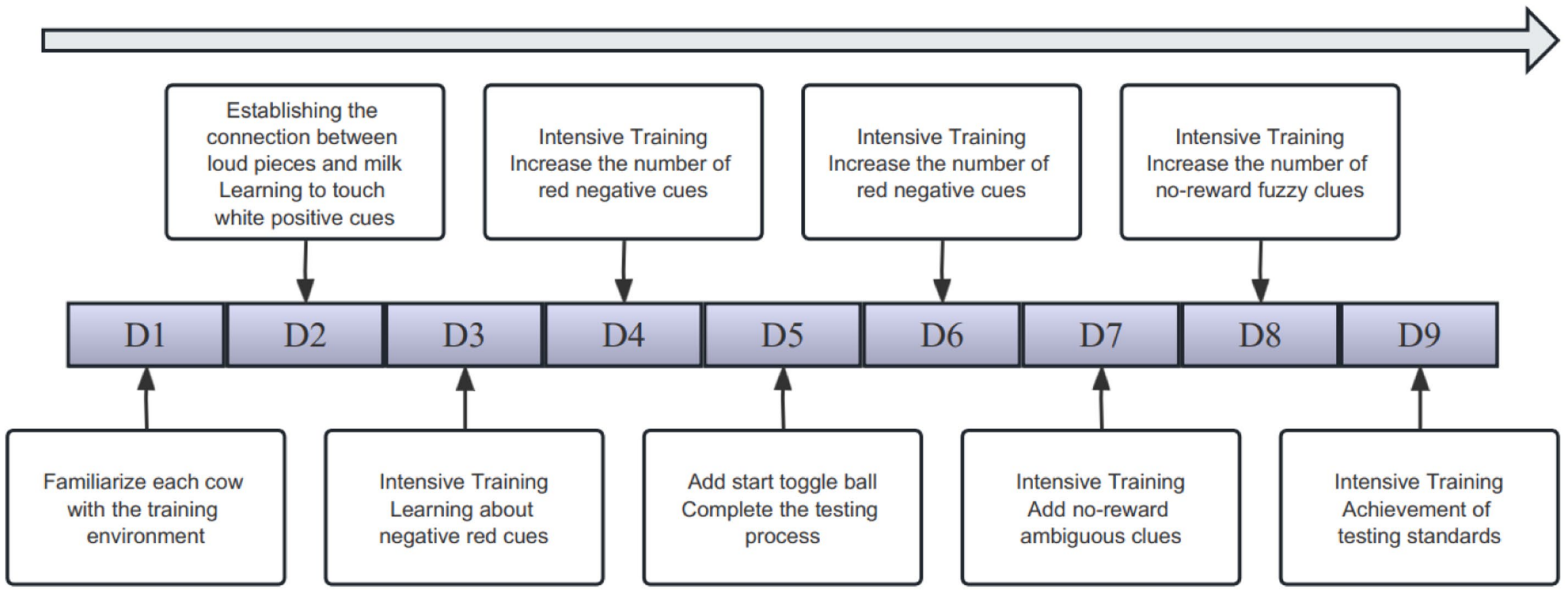
Simple flow chart of training.

The calves spent the first 10 min getting to know their new environment in the training enclosure while drinking 1.5 liters of milk from a bottle. The training sessions started on the fifth day and continued every day for 3 h, at 05:30, 12:30, and 17:30, when the milk was fed. The calves received 1.2 to 1.5 liters of milk during these sessions, which took place only in the assigned training enclosure and lasted for 10 to 35 min. A calf would immediately end the training session if it stood still for more than 2 min. Moreover, in order to reduce the possibility of dissatisfaction in the calves, we used a pseudo-randomized picture layout when introducing negative stimuli. The following is the training protocol:

- At the age of 5 days, the calves undergo a structured training routine. At 05:30, they are placed in the training pen to establish a connection between the rattle and feeding. The trainer presses the rattle twice consecutively to produce a sound, promptly followed by offering an appropriate amount of milk from a bottle. This process is repeated 25 times. At 12:30, the calves learn to associate touching a picture with a positive cue. A white picture is displayed, and the calf is gently guided to touch it. Upon contact, the trainer presses the rattle twice and immediately rewards the calf with a mouthful of milk. This sequence is repeated 25 times. At 17:30, the calves continue their training to touch the picture. A white picture is once again presented as a positive cue, and the calf is encouraged to touch it. Once the picture is touched, the rattle is pressed twice, and the calf is rewarded with another mouthful of milk. This training session is repeated as necessary to reinforce the learned behavior.
- At the age of 6 days, at 05:30, the calves underwent a training session. They were taught to respond to the sound of a rattle by touching a picture and then turning around to walk to a designated milk access point. This process was repeated ten times, with calves that failed to complete it receiving manual assistance up to three times before being allowed to try again independently. The session concluded once each calf had consumed 20 sips of milk. Later that day, at 12:30, the calves underwent further reinforcement of this training. They were again required to touch the picture, hear the rattle, and then walk back to the milk access point to drink the milk alone. This was repeated until each calf had consumed a total of 20 sips. At 17:30, a new element was introduced into the training: two red pictures were inserted among the 20 white pictures. If a calf touched a red picture, an audible “beep” sound was emitted as a negative cue. Calves that made this mistake were not given milk and were allowed three attempts to correct their behavior before receiving manual assistance.
- At the age of 7 days, the calf undergoes intensive training sessions. At 05:30, the number of red pictures inserted is increased to 4. The calf pauses for 5 s or longer at the red picture before switching to a white picture, which lights up. Upon touching the white picture, the calf receives a rattle and milk reward. At 12:30, the number of red pictures is further increased to 6, and at 17:30, it is raised to 10.
- At the age of 8 days, a start-switching ball is introduced at 05:30. When the calf enters the pen, the screen remains off. The calf must locate and touch the start-switching ball to activate the screen, revealing white pictures. Milk is rewarded after touching the pictures. During feeding, the screen is turned off again. This process is repeated until the calf has touched 20 white pictures, completing the training. At 12:30, the training is repeated until the calf can accomplish the task independently. At 17:30, the training progresses by adding 3 red pictures to the sequence of 20 white pictures. After touching the start-switching ball, the calf lights up a red picture. The calf is then guided to touch the ball again, switching the screen to display a white picture.
- At the age of 9 days, the calf undergoes further intensive training. At 05:30, the number of red pictures is increased to 6, superimposed on the white pictures. Gradually, the rattles are phased out, and no rattles are given after touching the white pictures. At 12:30, the number of red pictures is raised to 10, and at 17:30, it is increased to 15.
- At the age of 10 days, the 05:30 session involves 15 red pictures. During the 12:30 session, 3 non-rewarded pictures are added to the sequence of 20 red and 20 white pictures. These non-rewarded pictures resemble the white pictures but do not result in any reward or punishment. By the 17:30 session, the number of non-rewarded pictures is increased to 6.
- At the age of 11 days, the number of non-rewarded pictures continues to increase. The 05:30 training session includes 6 non-rewarded pictures, the 12:30 session has 10, and the 17:30 session features 15 non-rewarded pictures.
- At the age of 12 days, all three training sessions consist of 40 white pictures and 20 red pictures. Half of the white pictures are non-rewarded. The calves successfully complete all 60 pictures without making any errors (Figure 2).

### 2.3 Dehorning of calves

**Figure 3:**
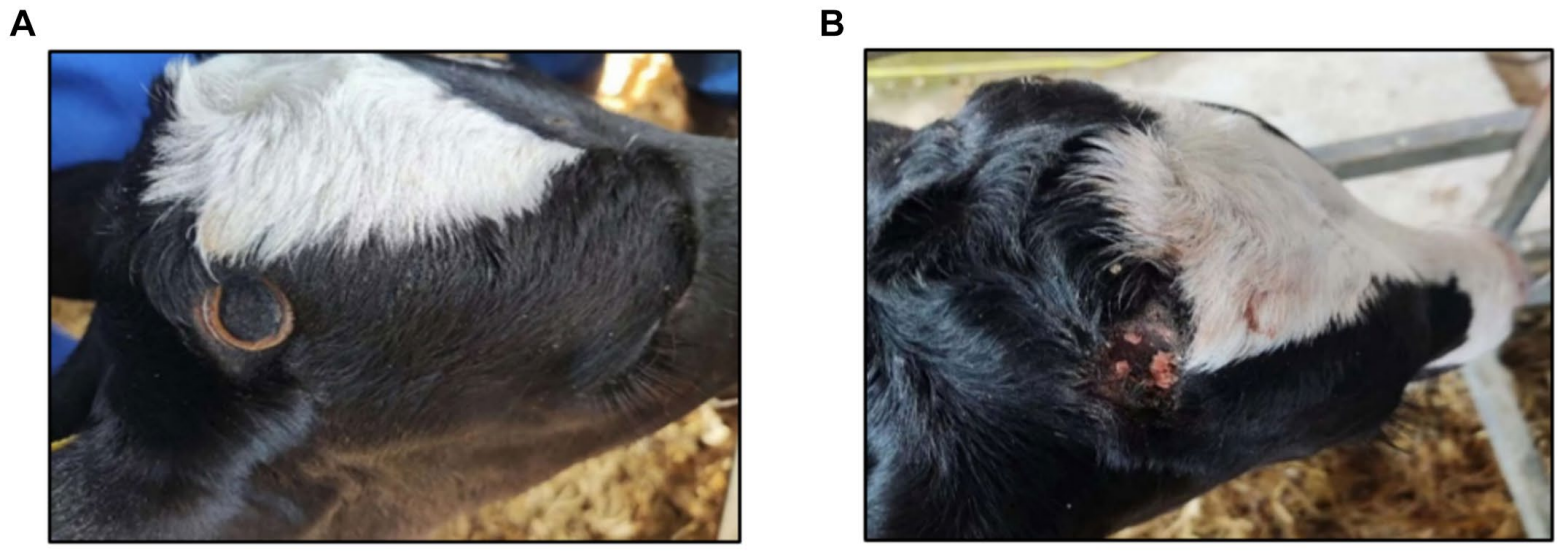
**(A)** Effect of calves dehorned by dehorning hot-iron. **(B)** Effect of calves dehorned by dehorning creams.

Dehorning was performed on calves at 13 days of age. Veterinarians, dressed in camouflage uniforms to distinguish them from other rearing personnel, followed the same order as during training. The DI group used the dehorning hot-iron to dehorn, and the dehorning equipment used was produced by QJQ500, Zhi Shepherd Company in China. The iron was heated to 600 ~ 800°C, placed firmly on the horn buds, and rotated until a bronze-colored ring appeared at the base, indicating successful dehorning in Figure 3A. The DC group was dehorned using the dehorning cream, which was produced by Dr. Naylor’s Company, United States (calcium hydroxide 38%, sodium hydroxide 21%, glycerol 19.8%, and water 21.2%). Hair around the horn buds was shaved, and a 2 cm diameter layer of cream was applied in Figure 3B. Calves were monitored for 10 min to prevent them from rubbing the cream off.

### 2.4 Behavioral records

**Figure 4:**
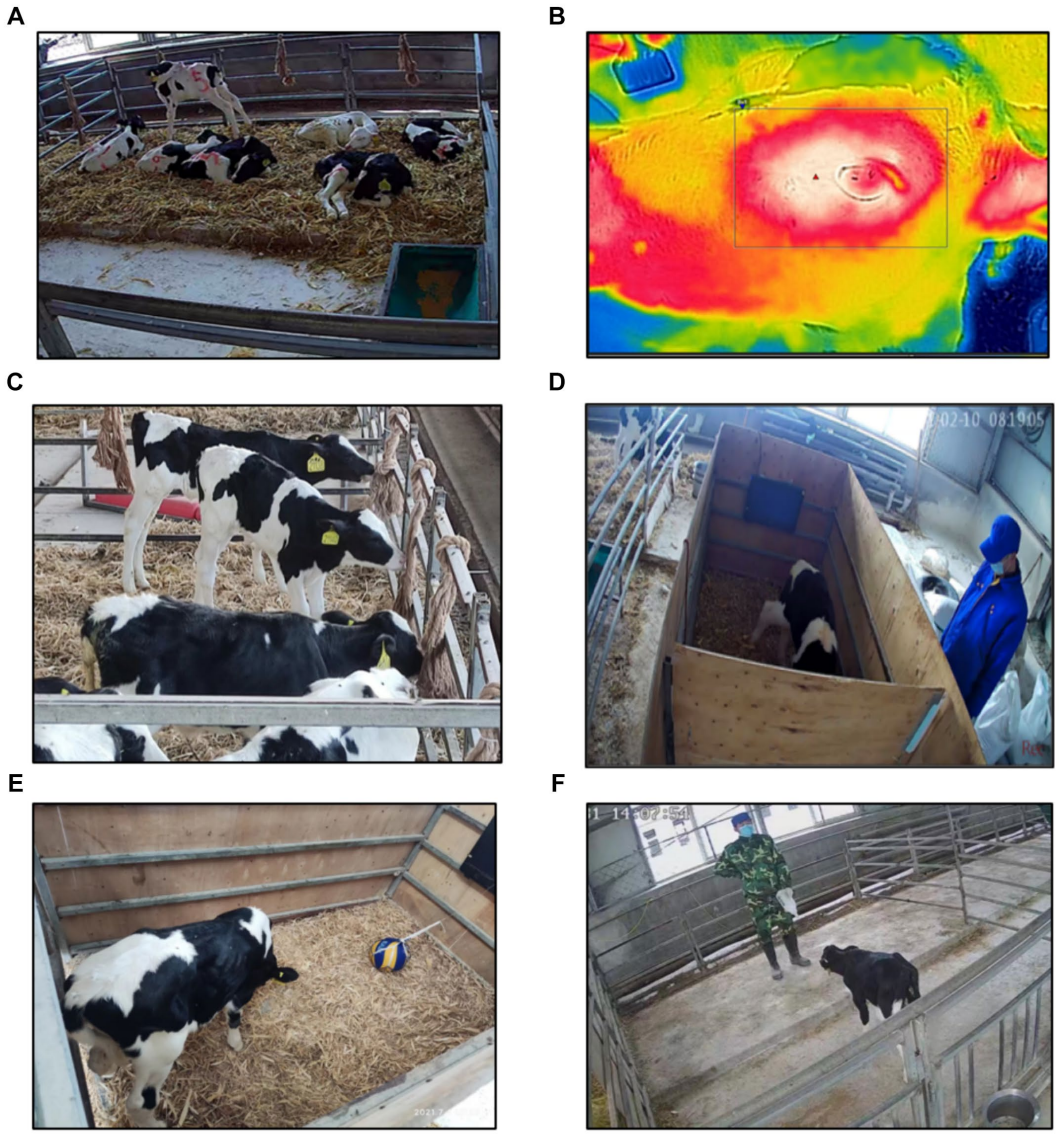
**(A)** Schematic of calf being tagged. **(B)** Schematic of FLIR tool recording calf’s eye temperature Figure. **(C)** Schematic of calf’s play behavior. **(D)** Schematic of judgment bias test. **(E)** Schematic of calf’s novelty stimulus Figure. **(F)** Schematic of calf’s dehorning man fear test.

At 11 days of age, for ease of viewing in the video, calves were marked with red crayon on their left side, right side, and white patch on their back, along with serial numbers in Figure 4A. Behavioral expressions were recorded using a camera (JDVISION, AD-132GE, China) mounted on the roof of the calf shed, capturing the entire area within the enclosure. Calves were video-recorded for 24 h before and after dehorning, focusing on lying time and changes in behavior (lying-standing transitions, ear-flicking, head-shaking, head-scratching, Top-rubbing, play behaviors). Definitions of these behaviors are provided in Table 1. Four skilled observers analyzed the videos, noting lying time every 5 min and continuously observing the other behaviors.

**Table 1:** Behavioral categories and definitions.

Behavior category	Definition
Lying	Lateral flank contact with the ground without any supportive force in the legs
Lying-Standing Transition	From standing to prone or from prone to standing
Head-Scratching	Contact of any part of the head with straw/fence/companion
Head-Shaking	Violent shaking of the head (head tossing)
Ear-Flicking	Rapid swinging of the ears
Top-rubbing	Cutting the head with the hind legs
Play Behavior	Calf licks twine with tongue

### 2.5 Physiological tests

#### 2.5.1 Eye temperature measurement

Eye temperature recordings were taken at 10 min before dehorning and at 10 min, 1 h, 3 h, 7 h, 22 h, 32 h, 48 h, and 72 h after dehorning. An infrared camera (C3-X, TELEDYNE FLIR, United States, IR 128 × 96, NETD <70 Mk, FOV 53.6^O^) operated by two individuals to minimize stress caused by human interaction, was utilized for thermal imaging of the calves’ eyes. Prior to measurement, the internal temperature of the infrared camera was calibrated to the ambient temperature. The camera was positioned at a right angle (approximately 90°) and at a distance of 0.5 m from the calf’s left eye to capture the region of interest. Subsequent to image acquisition, the FLIR tool (v4.1; FLIR Systems) was employed to analyze the captured images and determine the maximum eye temperature, typically located at the inner posterior eyelid margin or the lower eyelid’s tear punctum, as shown in Figure 4B. Eye temperature measurements had to be completed before collecting saliva and blood samples to avoid stress induced by other procedures.

#### 2.5.2 Serum sample collection

Serum samples were collected at 10 min before dehorning and at 50 min, 8 h, 24 h, 48 h, and 72 h after dehorning. Jugular venipuncture was performed with one person calming the calf and positioning it on its side to expose the neck, while another person collected the blood from the jugular vein into clot activator tubes. The procedure was conducted to minimize stress, and the blood draw was completed within 3 min. After collection, the blood was centrifuged at 3000 rpm for 10 min, and the serum was aliquoted into 1.5 mL Eppendorf tubes and stored at −20°C for subsequent analysis. All samples will be tested collectively following the completion of all experiments.

#### 2.5.3 Saliva sample collection

Saliva samples were collected at 30 min before dehorning and at 40 min, 3.5 h, 7 h, 24 h, 48 h, and 72 h after dehorning. Sterile saliva collection swab was created by tying a 1 g piece of defatted cotton to a 50 cm length of nylon rope. The prepared swab was placed in the calf’s mouth, and after the calf had chewed it thoroughly, the defatted cotton was removed and placed into a 20 mL disposable syringe that serves as the container. Within 10 min, the saliva absorbed in the swab was expressed into an Eppendorf tube, which was then stored at −20°C for subsequent cortisol analysis.

#### 2.5.4 Measurement of indicators

The serum physiological markers, including haptoglobin, substance P, and Interleukin-6 (IL-6), along with the salivary cortisol level, were assayed using the enzyme-linked immunosorbent assay (ELISA). All assays were performed according to the manufacturer’s instructions provided by FROM, United States.

### 2.6 Emotional testing

#### 2.6.1 Observation of play behavior

Figure 4C demonstrates the design of the calf toys used in our study. These toys consisted of 28 mm diameter, 1.2 m long hemp ropes, each with spikes approximately 20 cm in length knotted at both ends. These toys were folded and securely hung at 1.2 m intervals above the fence opposite the entrance, maintaining a 60 cm gap between the ropes and both ends of the fence. Additionally, the side fences had ropes fixed at 2 m intervals, creating a 1 m gap between the ends. Altogether, six fixed ropes were provided for the calves to engage in play. After dehorning, the calves’ play behavior was continuously monitored through video footage for 48 h. During this period, we focused on recording the play time during the four sites in 2 days (After dehorning 6–8 h, 18–20 h, 30–32 h, 42–44 h) when play behavior typically peaks (37).

#### 2.6.2 Judgment bias test

The design of this section references Heather’s Institute design (32). As depicted in Figure 4D, calves underwent judgment bias tests at specific intervals: 6 h before dehorning and 1, 7, 25, and 31 h after dehorning. The test consisted of randomly presenting 60 solid-color pictures, including 24 white and 24 red images, and 4 images each of red with varying saturations (60, 40, and 20%). All pictures exhibited uniform contrast, sharpness, and brightness when displayed. A calf’s interaction with the start-up switching sphere during the test was recorded as a touch. Positive reinforcement (milk feeding) was given for touching white pictures, while negative reinforcement (light blow to the head) was administered for touching red pictures. Touches on other pictures had no consequence.

Before commencing the test, monitoring equipment was activated for recording purposes. Once the calf was guided into the training pen, the PowerPoint presentation was initiated to measure the calf’s reaction time to the 60 pictures. The test was terminated if the calf exhibited no touches for over 3 consecutive minutes or displayed lying behavior before its conclusion. To ensure minimal distractions, the testing environment was maintained in silence. The total time required for the test and the time needed to respond to 60, 40, and 20% degraded cues were compared between the two groups of calves.

#### 2.6.3 Novelty stimulus test

Following the judgment bias test, as illustrated in Figure 4E, the calf remained in the training pen while the screen was turned off. A novel object (a volleyball unfamiliar to the calf) was carefully positioned 50 cm from the calf’s front. Simultaneously, the calf’s behavior was video-recorded for 5 min, maintaining silence throughout the test. The video captured the calf’s reaction delay to the novel object, measured from the moment it was introduced in the training enclosure until the calf interacted with it. The time required for both groups of calves to respond to novel stimulus was compared as the final assessment.

#### 2.6.4 Dehorner fear test

As shown in Figure 4F, an empty pen identical to the feeder pen served as the testing area. To mitigate the influence of the testing environment, calves were acclimated to the testing area for 10 min on the day prior to the experiment. Tests were conducted 2 and 6 h after dehorning. During the test, the dehorner stood 3 m from the entrance, holding the feeder at knee level to guide the calf into the empty pen. Timing commenced when the calf’s entire body was inside the pen. The response delay time, from the calf’s entrance into the pen until its first contact with the dehorner, was recorded for a duration of 5 min. The time taken by the calves in both groups to respond to the dehorner was compared as the final measure.

### 2.7 Statistical analysis

SPSS 19.0 was used for statistical analysis. Beforehand, all experimental data underwent a normality test (Shapiro). For non-normally distributed data, non-parametric tests (Wilcoxon) were employed.

In our analysis, “behavior” served as the dependent variable, while the dehorning method and time were treated as fixed factors. The lying down time was matched with the day preceding dehorning, analyzed using a paired *t*-test. Other behaviors were subjected to multiple group comparisons using LSD in multifactor ANOVA. The relationship between behavior and time was delineated using a nonlinear regression model optimized by a cubic polynomial. Regression equations, scatter plots, and curves were derived from the processed data, selecting the best-fit regression curves.

Affective tests, specifically the novelty and dehorner fear tests, were analyzed with the main-Whitney test for nonparametric data. Game behavior was compared to pre-dehorning baseline values using paired *t*-tests, while independent *t*-tests assessed dehorning method comparisons. The judgment bias test considered the time taken to touch a component, normalizing it by dividing with the baseline time and then analyzed using an independent *t*-test.

Physiological indicators underwent multifactorial ANOVA, treating them as dependent variables, with dehorning method, time, and initial data as covariates. This comprehensive approach analyzed the impact of dehorning methods, time effects, and their interactions. Multiple group comparisons employed the LSD of multifactor ANOVA. When examining time effects, post-dehorning physiological values were benchmarked against pre-dehorning ones.

Data presentation followed the “Mean ± Standard Error” (Mean ± SEM) format. GraphPad Prism 9.0 facilitated the visualization of our findings. Significant differences are denoted as follows: “*” for the DI group versus the pre-dehorning period, “#” for the DC group versus the pre-dehorning period, and “+” for comparisons between the DI group and DC groups. One “*” indicates *p* < 0.05 two “**” indicates *p* < 0.01, three “***” indicates *p* < 0.001, four “****” indicates *p* < 0.0001, but in the textual expression *p* value are all expressed as “0.05”.

## 3 Results

### 3.1 Effect of different dehorning methods on calf behavior

#### 3.1.1 Lying time

**Figure 5:**
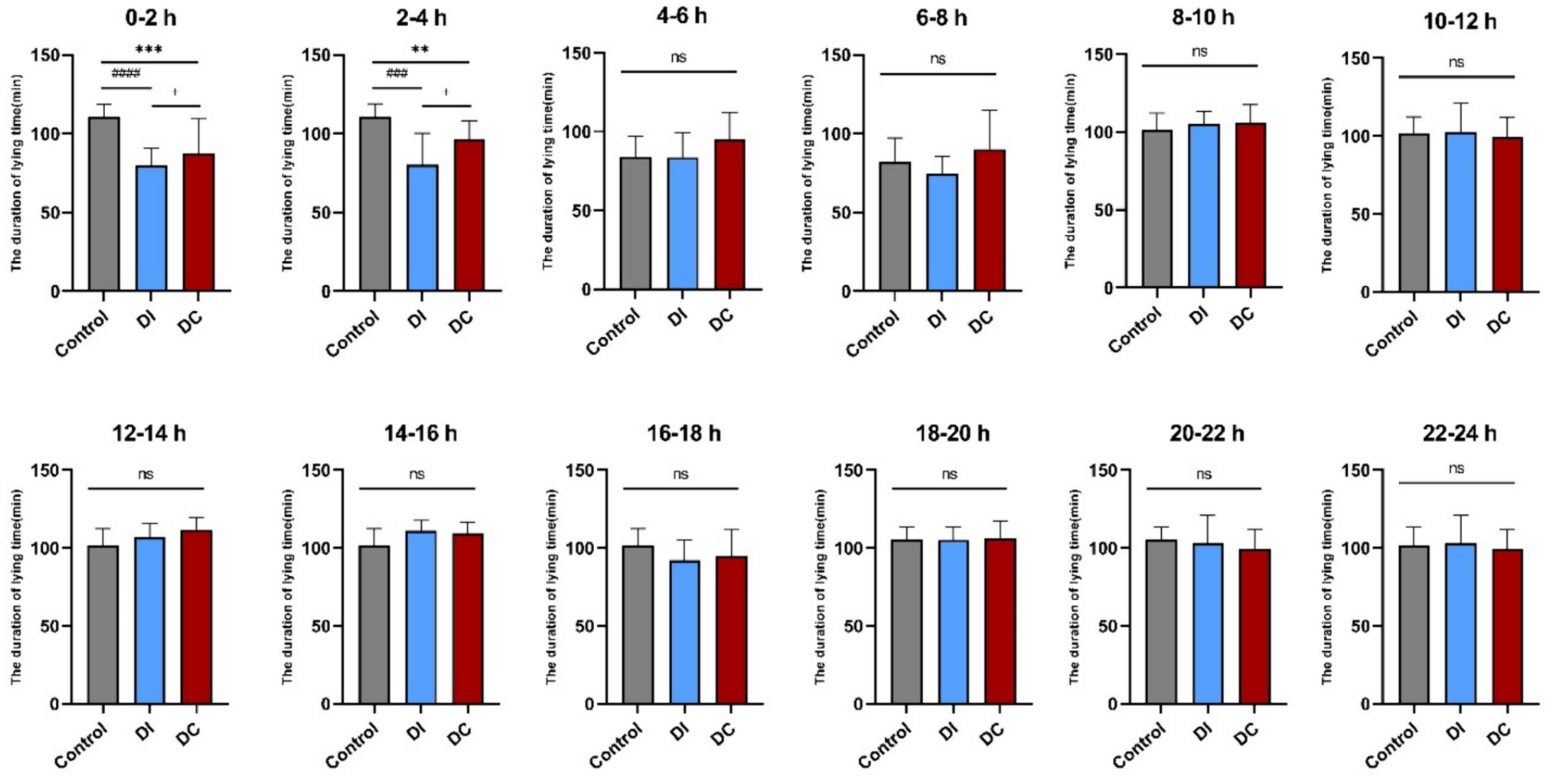
Effect of different dehorning methods on calf lying time. Control, before dehorning; DI, dehorning iron group; DC, dehorning cream group. “*” indicates significant difference in the DC group compared to the pre-dehorning state; “#” indicates significant difference in the DI group compared to the pre-dehorning state; “+” indicates significant difference between the DI and DC groups.

As Figure 5 illustrates, a noteworthy reduction in lying time was observed in both the DI and DC groups immediately after dehorning (0–2 h and 2–4 h), in contrast to the pre-dehorning period (DC: 0–2 h *p* < 0.001, 2–4 h *p* = 0.0053; DI: 0–2 h *p* < 0.0001, 2–4 h *p* < 0.001). Notably, the DC group exhibited a significantly longer lying time compared to the DI group during these initial hours (0–2 h *p* = 0.038, 2–4 h *p* = 0.026). However, this difference diminished as time progressed, with no significant changes observed between the two groups from 4 to 24 h post-dehorning when compared to the pre-dehorning and inter-group periods (*p* > 0.05).

#### 3.1.2 Number of lying-standing transitions

**Figure 6:**
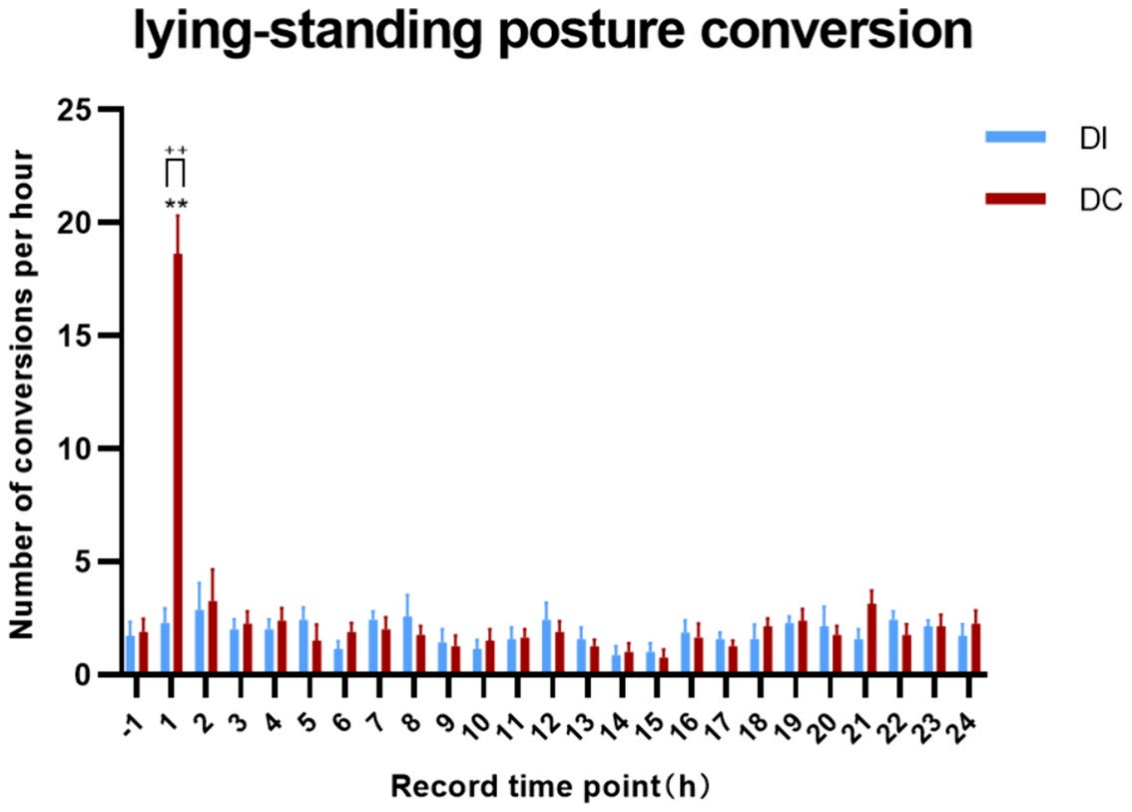
Effects of different dehorning methods on calf lying-standing transitions behavior. Control, before dehorning; DI, dehorning iron group; DC, dehorning cream group. “*” indicates significant difference in the DC group compared to the pre-dehorning state; “+” indicates significant difference between the DI and DC groups.

According to Figure 6, the DI group showed no significant variation in the number of lying-standing transitions within 24 h of dehorning when compared to the pre-dehorning period (*p* > 0.05). On the other hand, the DC group exhibited a marked increase in lying-standing transitions 1 h after dehorning (*p* = 0.0035), which was also significantly higher than that observed in the DI group (*p* = 0.0082).

#### 3.1.3 Head-scratching behavior

**Figure 7:**
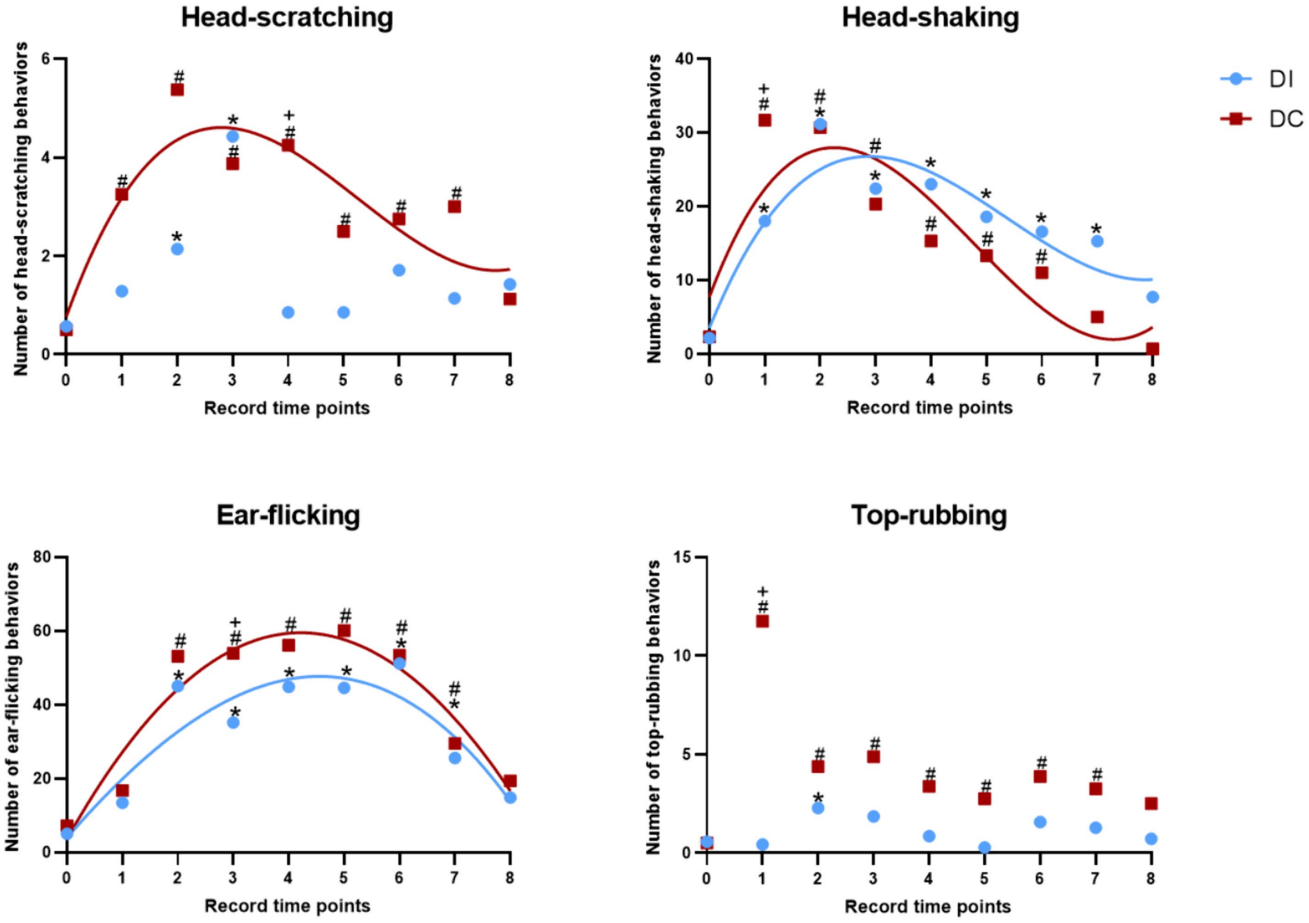
Effects of different dehorning methods on calf behavior. DI, dehorning iron group; DC, dehorning cream group. “*” indicates significant difference in the DC group compared to the pre-dehorning state; “#” indicates significant difference in the DI group compared to the pre-dehorning state; “+” indicates significant difference between the DI and DC groups.

As depicted in Figure 7, both the DI and DC groups demonstrated a significant increase in head-scratching behavior within 8 h of dehorning when compared to the pre-dehorning period (*p* < 0.05). Specifically, the DI group showed a notable rise at 2 h and 3 h post-dehorning, while the DC group exhibited a consistent increase throughout the observed period. Interestingly, the DC group displayed a significantly higher frequency of head-scratching compared to the DI group at 4 h after dehorning (*p* = 0.037). Additionally, the regression curve fit was found to be better in the DC group (*p* = 0.015).

#### 3.1.4 Head-shaking behavior

As illustrated in Figure 7, within the first 24 h after dehorning, both the DI and DC groups exhibited a significant increase in head-shaking behavior compared to pre-dehorning levels (*p* < 0.05). Specifically, the DI group showed a marked increase in head-shaking from 1 to 7 h post-dehorning, while the DC group demonstrated a significant increase from 1 to 6 h post-dehorning (*p* < 0.05). Notably, the DC group displayed significantly higher levels of head-shaking than the DI group at 1 h after dehorning (*p* = 0.032). The regression curves for both groups indicated a good fit (DI: *p* = 0.016, DC: *p* = 0.044).

#### 3.1.5 Ear-flicking behavior

According to Figure 7, both the DI and DC groups showed a significant increase in ear-flicking behavior within 8 h of dehorning compared to pre-dehorning levels (*p* < 0.05). In particular, the DI group exhibited a notable increase in ear-flicking from 2 to 7 h post-dehorning, while the DC group displayed a significant increase from 2 to 7 h post-dehorning as well. Interestingly, the DC group demonstrated significantly higher levels of ear-flicking than the DI group at 3 h after dehorning (*p* = 0.001). The regression curves for both groups provided a good fit (DI: *p* = 0.001, DC: *p* = 0.001).

#### 3.1.6 Top-rubbing behavior

As presented in Figure 7, within 8 h of dehorning, the DI group showed a significant increase in top-rubbing behavior at 2 h post-dehorning compared to pre-dehorning levels (*p* < 0.05). Similarly, the DC group exhibited a significant increase in top-rubbing behavior from 2 to 7 h post-dehorning compared to pre-dehorning levels (*p* < 0.05). Additionally, the DC group displayed significantly higher levels of top-rubbing behavior than the DI group at 1 h after dehorning (*p* = 0.028) (Table 2).

**Table 2:** Fitting curves of the effects of different dehorning methods on calf behavior.

Behavior	Method	Regression equation	*R*^2^	*p*-value
Head-scratching	Iron	y = 0.0263x3 − 0.4063x2 + 1.6275x + 0.4839	0.3378	0.447
	Cream	y = 0.0297x3 − 0.5632x2 + 2.6139x + 0.9442	0.8033	0.015
Head-shaking	Iron	y = 0.2703x3 − 4.3579x2 + 18.385x + 3.521	0.8513	0.016
	Cream	y = 0.4057x3 − 5.8121x2 + 20.017x + 7.7273	0.7771	0.044
Ear-flicking	Iron	y = 0.2037x3 − 4.6559x2 + 26.741x + 0.2592	0.8584	0.001
	Cream	y = 0.2888x3 − 6.3241x2 + 35.088x + 0.4588	0.9224	0.001
Top-rubbing	Iron	y = 0.0164x3 − 0.2357x2 + 0.9098x + 0.443	0.2242	0.709
	Cream	y = 0.0952x3 − 1.2229x2 + 3.7451x + 3.1654	0.2827	0.613

### 3.2 Effect of different dehorning methods on physiological indices of calves

#### 3.2.1 Effect on calf eye temperature

**Figure 8:**
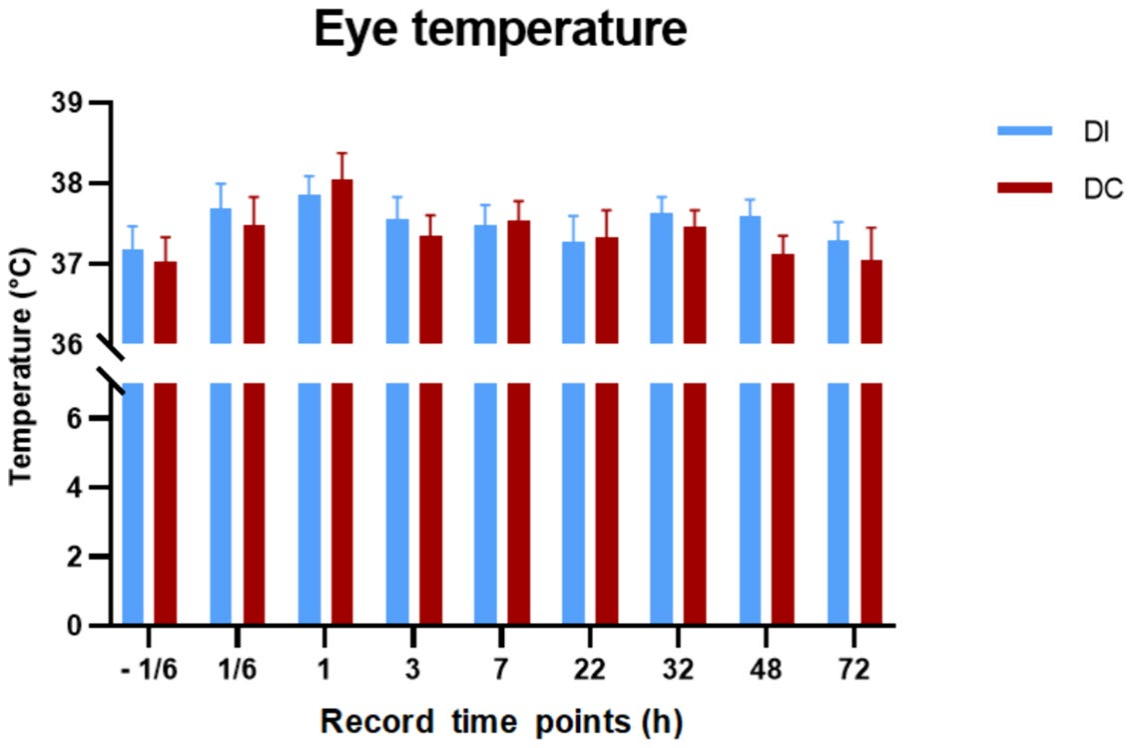
Effects of different dehorning methods on calves’ eye temperature. DI, dehorning iron group; DC, dehorning cream group.

As illustrated in Figure 8, there were no significant differences in eye temperature observed in calves before and after dehorning in both the DI and DC groups (*p* > 0.05). Additionally, the comparison between the two groups revealed no noteworthy changes in eye temperature (*p* > 0.05).

#### 3.2.2 Hormone level

**Figure 9:**
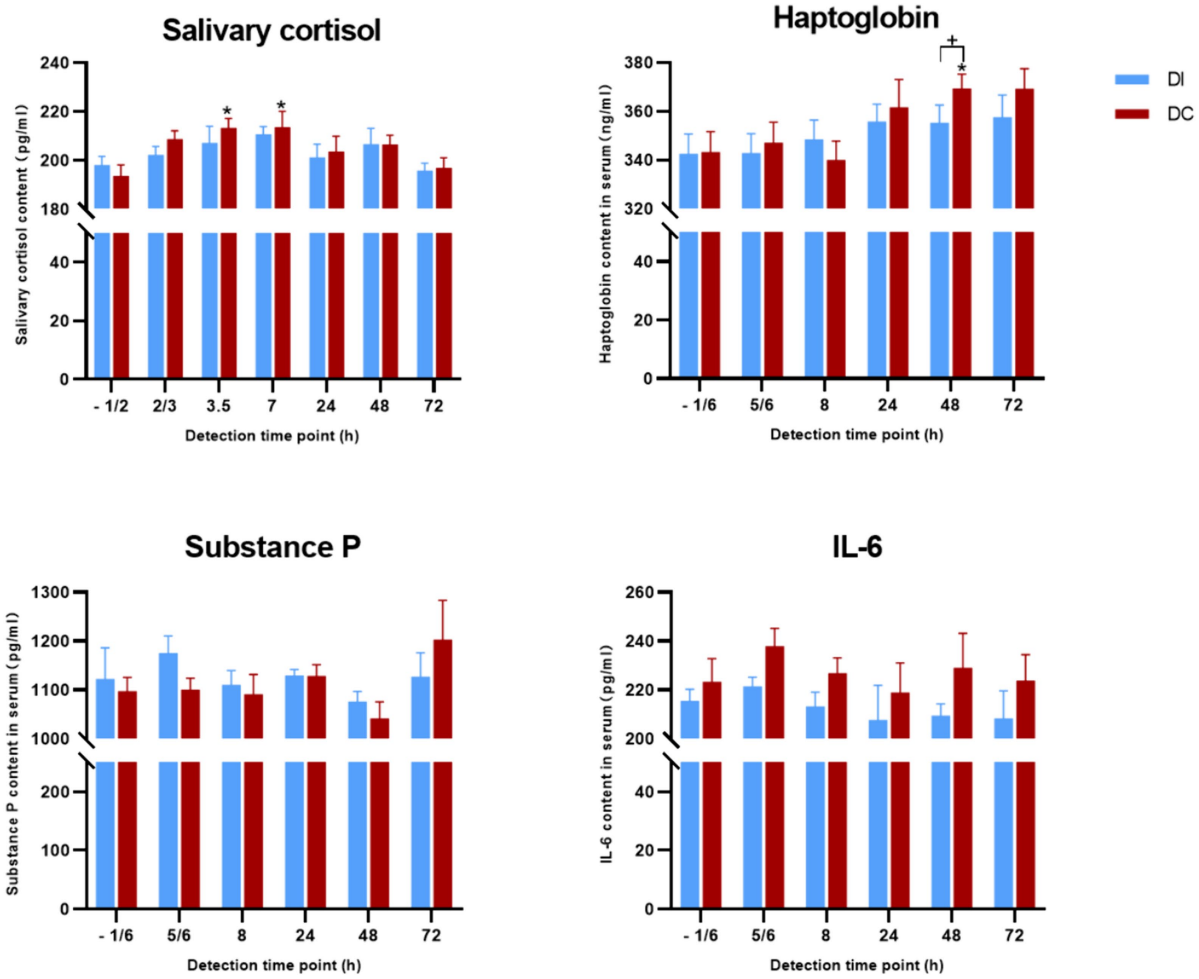
Effects of different exfoliation methods on physiological indexes. DI, dehorning iron group; DC, dehorning cream group. “*” indicates significant difference in the DC group compared to the pre-dehorning state; “+” indicates significant difference between the DI and DC groups.

Figure 9 demonstrates that the salivary cortisol, haptoglobin, serum substance P, and IL-6 levels in the DI group calves remained largely unchanged following dehorning (*p* > 0.05). However, in the DC group, salivary cortisol levels were notably elevated at 3.5 h and 7 h post-dehorning (*p* = 0.018, *p* = 0.043), while haptoglobin levels saw a significant increase 48 h after the procedure (*p* = 0.015). No significant differences were observed in serum substance P and IL-6 levels (*p* > 0.05). Furthermore, no appreciable disparities in these hormone levels were found between the two experimental groups (*p* > 0.05).

### 3.3 Emotional part

#### 3.3.1 Play behavior

**Figure 10:**
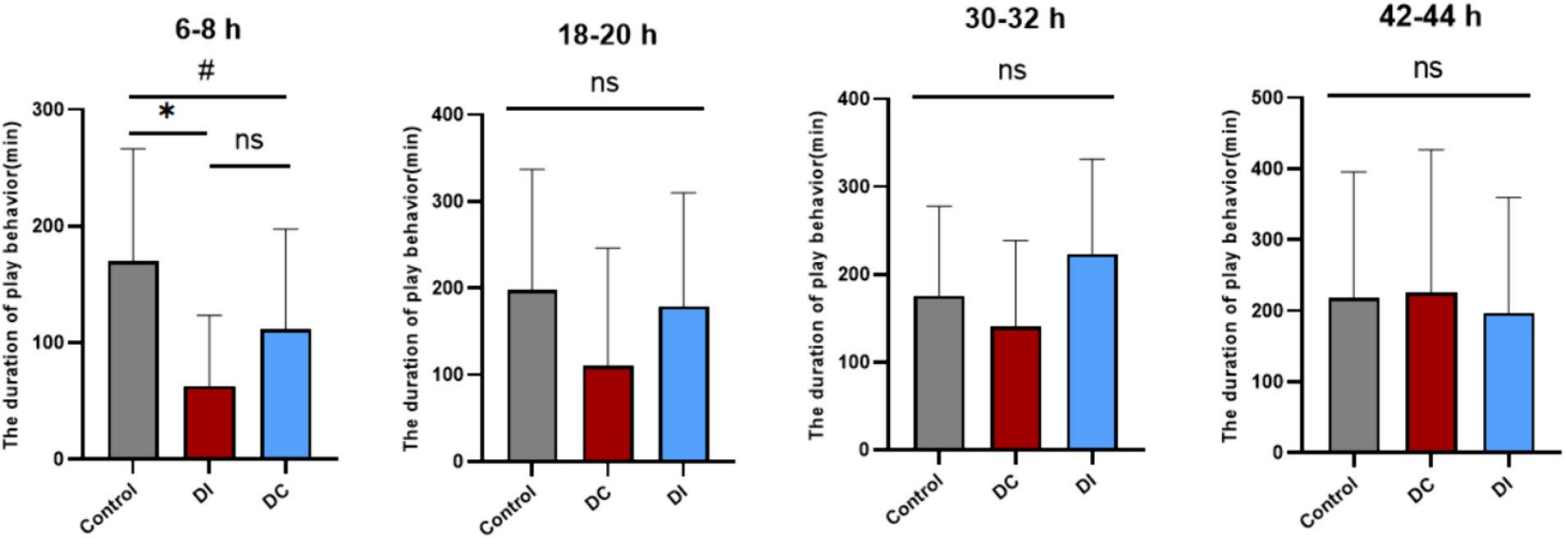
Effects of different dehorning methods on the duration of play behavior of calves. Control, before dehorning; DI, dehorning iron group; DC, dehorning cream group. “*” indicates significant difference in the DC group compared to the pre-dehorning state; “#” indicates significant difference in the DI group compared to the pre-dehorning state.

As Figure 10 illustrates, the play behavior of calves in both the DI and DC groups significantly decreased (DI: *p* = 0.012, DC: *p* = 0.02) during the 6–8 h period after dehorning compared to the corresponding time before the procedure. However, no notable difference was observed in the duration of play behavior between the two groups (*p* > 0.05). At other times, the play behavior of the experimental groups remained largely unaffected (*p* > 0.05).

#### 3.3.2 Judgment bias test

**Figure 11:**
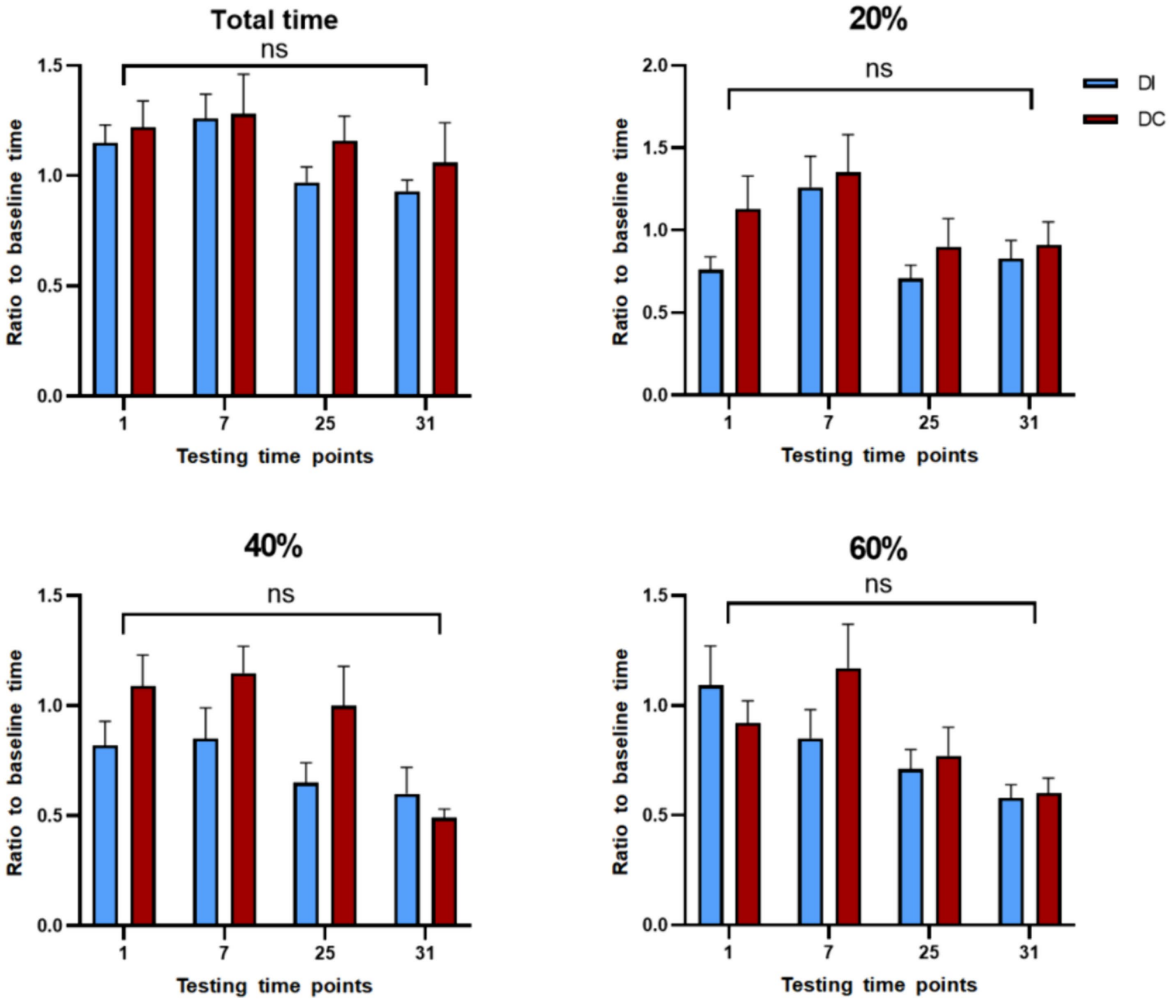
Effect of different dehorning methods on reaction time of judgment bias test. DI, dehorning iron group; DC, dehorning cream group.

According to Figure 11, there were no significant differences (*p* > 0.05) between the DI and DC groups in terms of total test time and reaction time to 20, 40, and 60% saturated red images after dehorning. Although the average total time for the DC group was higher than that of the DI group, the difference was not statistically significant (*p* > 0.05). Seven hours after dehorning, the DC group showed an increase in reaction times, but this trend was not statistically significant (*p* > 0.05). Meanwhile, the DI group demonstrated a gradual decrease in reaction time only for the 60% saturation red image group, while the other groups exhibited a similar increase as the DC group.

#### 3.3.3 Novelty stimulus test and dehorner fear test

**Figure 12:**
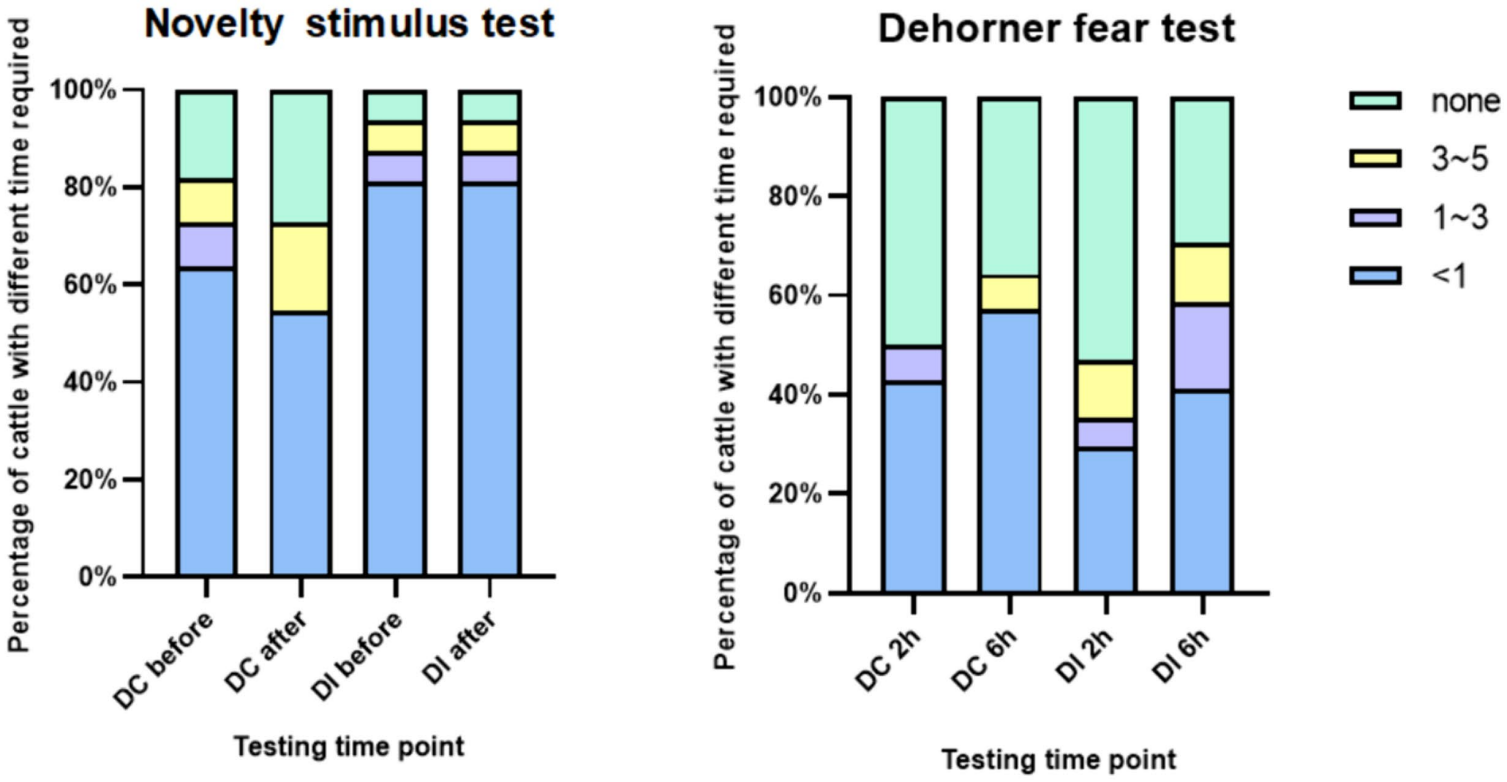
Reaction time of calves to novelty and dehorner.

As depicted in Figure 12, no significant differences (*p* > 0.05) were observed between the two experimental groups in their exposure to novelty stimulus across various time points, relative to the pre-dehorning period. Furthermore, there were no significant differences (*p* > 0.05) in the groups’ responses to the presence of the dehorner at two distinct time points (2 h and 6 h) following dehorning.

## 4 Discussion

The results of this study showed that following dehorning, calves in both groups significantly reduced the amount of time they spent lying down and increased their behaviors connected to discomfort. These results imply that the calves experienced discomfort in response to both dehorning methods. These findings suggest that calves’ behavioral responses to dehorning may be procedure-specific, with the location of tissue damage subject to ongoing injury. The stimulation of inflammatory mediators results in continuous impulses at the site of injury, leading to alterations in behavioral patterns (38). In a similar vein, Sutherland et al. noted that in dehorned calves, lying behavior decreased as a sign of pain (39). Additionally, our study showed that 1 hour after dehorning, the DC group had a considerably larger number of lying-standing transitions than the DI group. This suggests that although cauterization results in a painful experience that is more rapid but shorter-lived, the application of dehorning cream leads to a longer-lasting and slower (40). Prior research has indicated that dehorning causes calves to exhibit increased head-scratching, head-shaking, and ear-flicking behaviors, which eventually revert to their pre-dehorning levels (41). While it has been demonstrated that local anesthesia might lessen pain behaviors during dehorning, once the anesthetic effect wears off, these behaviors become more frequent (42). The calves in our study were not given any analgesia treatment to relieve their discomfort, and the notable behavioral alterations that followed dehorning suggest that both procedures were painful for the animals. Note that 1 hour after dehorning, the DC group exhibited considerably more top-rubbing activity. This might be attributed to either a burning sensation from the dehorning cream or a foreign body sense from the cream covering the horn buds.

Pain is one of the primary indicators of inflammation, and the inflammatory response associated with pain arises from sudden or prolonged exposure to noxious stimuli (43). Due to the interaction between pro-inflammatory mediators and nociceptors, the core and surface temperatures at the site of injury increase. At the site of inflammation, pro-inflammatory factors can cause vasodilation, increasing blood flow and vascular permeability, and releasing chemical mediators, leading to symptoms such as redness, swelling, pain, loss of function, and localized heat (44). It has been suggested that variations in calves’ eye temperatures can serve as a sign of severe pain (45), temperature variations can be measured using IRT after a nociceptive stimulus due to the activation of the nervous system and its vasomotor consequences, inducing an inflammatory process and an increase or decrease in heat radiation (26, 46). In our experiment, following dehorning, the ocular temperatures of both groups of calves increased, although the difference was not statistically significant. After 1 hour, the eye temperatures started to drop, indicating that the calves were still feeling the effects of the dehorning discomfort at this point. The age of the calves or the timing of the ocular temperature measurements may have contributed to the lack of a noticeable rise in eye temperature he DC group saw a larger change in ocular temperature than the DI group, suggesting that dehorning cream was more painful for the calves than dehorning hot-iron. In conclusion, calves respond to both dehorning methods with pain, but that dehorning cream causes a more severe and sustained pain response. These findings emphasize the need for less painful and more compassionate substitutes for the conventional dehorning techniques used in calf husbandry. Moreover, its non-invasiveness and the objective nature of its readout make it potentially very valuable.

Cortisol concentration variations are often a sign of the body’s physiological stress reactions (47). In the current study, which compared two ways of dehorning calves, it was shown that the application of dehorning cream caused cortisol levels to rise significantly at 3.5 and 7 h after dehorning, then gradually decline to pre-dehorning levels. On the other hand, there was a non-significant upward trend in the dehorning hot-iron group. These results are consistent with other studies by Stilwell et al. (48), which found that using a comparable cream, cortisol concentrations were higher three and 6 hours after dehorning. These findings point to a significant effect of dehorning on calves’ cortisol levels.

No discernible changes were seen in either group 24 h following the dehorning process when looking at the impact on haptoglobin, a possible indicator for tissue damage. These findings are in similar to a prior study conducted by Hempstead et al. (49). The dehorning cream group did, however, show a change in haptoglobin content after 48 h, suggesting that the cream may have induced discomfort that persisted for up to 48 h. However, some studies has also shown that no changes in the amounts of conjugated haptoglobin following the use of a dehorning cream on goats (14, 49). Regardless of the use of painkillers, substance P, a neuropeptide linked to pain, tension, and anxiety, did not show any appreciable variations in concentration following dehorning (24, 50). Additionally, Dockweiler et al. discovered that substance P levels were dependent on the age of the calf during dehorning, with lower amounts seen in calves that were 8 weeks old as opposed to those that were 6 months old (51). These results imply that substance P might not be a good predictor of pain during dehorning in young calves. Lastly, both groups’ levels of interleukin-6 (IL-6), a crucial component of immunological and inflammatory responses, rose 1 h after dehorning (52). IL-6 concentrations were higher in the dehorning cream group than in the dehorning hot-iron group, which may suggest that the calves felt more discomfort.

Dehorning calves has been demonstrated in the past to considerably lessen their play behavior (53, 54). These observations are consistent with our results, which show that calves’ playtime significantly decreased 6–8 h following dehorning. This shows that the calves’ positive affectivity was adversely influenced by both techniques we utilized in our study. A study has been shown that calves can pick up basic color discrimination tasks (55). When presented with a significant negative ambiguity cue (75% saturated red photos), calves in our study first displayed insensitivity. We adjusted the saturation level of the cue to progressively increase its fuzziness in order to remedy this. This method is predicated on the idea that when an ambiguous cue is shown to animals frequently and they grow to associate it with a certain result, they may lose that ambiguity (56). Unrewarded ambiguous signals might become less salient during training if they receive partial reinforcement (57). During the testing phase, we implemented this approach by lowering the percentage of each ambiguous cue to 6.66% and depriving calves of rewards after they touched the white graphic, which represents the positive signal.

According to our research, following the dehorn, calves in both dehorned groups did not exhibit a decreased reactivity to positive signals. Their constant touching of all white photos and maintenance of the anticipation of positive cues during testing were clear. This implies that the calves’ incentive to acquire incentives did not change even though dehorning had a detrimental effect on their playful behavior. In order to identify emotional changes brought on by unpleasant procedures, Lecorps et al. have investigated the use of reaction time to ambiguous stimuli as an indication of judgment bias testing, found an increase in reaction time to highly favorable ambiguous stimuli after dehorning 7 hours (58). However, the reason for the non-significant difference in the present study, despite an upward trend being observed, may be that our calves were younger and less responsive at the time of dehorning compared to 35-day-age. An uncomfortable process caused the calves to experience negative effect, and a battery of tests was performed at 1, 7, 25, and 31 h following dehorning in order to ascertain the duration of pain induced by the two dehorning methods. In comparison to the dehorning hot-iron (DI) group, the dehorning cream (DC) group’s total test length was longer, indicating that the dehorning cream’s high persistence may have contributed to the group’s ongoing discomfort.

Pain can cause fear in animals, which in turn affects their response to unfamiliar situations or people (54). Therefore, animals in pain are not willing to touch new experiences and strangers (59). In both the novelty stimulus and dehorner fear test conducted in this study, there was no significant difference in the response time of calves before and after dehorning to novelty stimulus and fear test. Additionally, these response times did not change over time, and there was no significant difference in response times between the two groups. The reason for this may be that the pain caused by dehorning in calves did not reach a level that would affect their exploratory behavior. The calves in this study were low day-age and raised in groups, which can enhance their stress resilience (55). This could be another reason why there was no difference in the response of calves to novel objects and the dehorning stimulus in this study.

Additionally, this study has its limitations, such as the lack of objective pain assessment through techniques like facial expression analysis or heart rate variability (60, 61). Additionally, detailed pain assessments could be conducted using acute pain scales validated by certain researchers (62). Finally, the evaluation could also involve the use of analgesic treatments during the dehorning process (63, 64). Future research could enhance the comprehensive pain assessment system to address these gaps.

## 5 Conclusion

The comparison of two dehorning types in terms of pain induction in calves suggests that dehorning cream led to significantly higher pain-related behaviors and less playing in calves compared to hot-iron dehorning. Additionally, the dehorning cream increased the levels of salivary cortisol and haptoglobin, further highlighting the pain response in calves. When comparing the two methods, the dehorning cream appeared to cause more pronounced pain-related behaviors in calves. Therefore, dehorning with hot-iron is a relatively better choice for reducing pain in calves, in order to minimize the discomfort experienced by young calves during the dehorning process. The calves of low day-age in present study exhibited a brief and weak pain response to dehorning, which suggesting that dehorning earlier may be preferable, indicating a need for further research on the topic.

## Data availability statement

The original contributions presented in the study are included in the article/supplementary material, further inquiries can be directed to the corresponding author.

## Ethics statement

The animal study protocol was approved by the Science and Technology Ethics Committee of Heilongjiang Bayi Agricultural University (Ethics code: DWKJXY2022034). The studies were conducted in accordance with the local legislation and institutional requirements. Written informed consent was obtained from the owners for the participation of their animals in this study.

## Author contributions

WC: Conceptualization, Methodology, Validation, Writing – original draft. ML: Conceptualization, Data curation, Formal analysis, Writing – original draft. TG: Formal analysis, Methodology, Software, Writing – original draft. SZ: Investigation, Writing – review & editing. GY: Conceptualization, Resources, Supervision, Validation, Writing – review & editing.
